# Engineering Active Micro and Nanomotors

**DOI:** 10.3390/mi12060687

**Published:** 2021-06-11

**Authors:** Mingwei Liu, Kun Zhao

**Affiliations:** Frontier Science Center for Synthetic Biology and Key Laboratory of Systems Bioengineering (Ministry of Education), School of Chemical Engineering and Technology, Tianjin University, Tianjin 300072, China; mingweiliu@tju.edu.cn

**Keywords:** self-propulsion, micromotors, Janus particles, microjets

## Abstract

Micro- and nanomotors (MNMs) are micro/nanoparticles that can perform autonomous motion in complex fluids driven by different power sources. They have been attracting increasing attention due to their great potential in a variety of applications ranging from environmental science to biomedical engineering. Over the past decades, this field has evolved rapidly, with many significant innovations contributed by global researchers. In this review, we first briefly overview the methods used to propel motors and then present the main strategies used to design proper MNMs. Next, we highlight recent fascinating applications of MNMs in two examplary fields, water remediation and biomedical microrobots, and conclude this review with a brief discussion of challenges in the field.

## 1. Introduction

Small-scale living organisms are capable of converting diverse types of energy into mechanical work. In order to sustain the basic functions of cell and adapt to environmental changes, they have evolved delicate biomolecular motors which are responsible for energy conversion and movement occurring at both a molecular and a macroscopic scale in many organisms [1]. On the other hand, humans’ technology also advances quickly and we can harness different types of power sources and convert them into mechanical work more skillfully. As our ancestors could only drive cattle or horses for work in ancient times, in the modern era we can now make a variety of efficient machines like combustion engines and electric motors and use them to change the world. Particularly, at the microscopic scale, micro- and nanomotors (MNMs), whose typical sizes are in a range of 100 nm ~100 µm, have been continuously developed to help people explore this ‘micro world’.

MNMs are small autonomous devices capable of performing complex tasks while being self-propelled in fluids [2,3,4,5,6,7,8,9,10,11,12,13]. They can convert energy into motion by consuming fuels in solutions or by absorbing power from external sources such as ultrasound [14], light, thermal fields [15], magnetic fields [16], etc., and thus are able to transport mass to specific destinations at the micro/nano scale [6]. This gives them great potential in numerous applications such as nanotechnology [9,10], biomedical engineering [12,13], and environmental engineering [2,17], etc.

However, MNM devices are different from those machines built at the macroscopic scale for the following three reasons [18,19]: (i) The Reynolds number (Re) for most cases is often small, which means inertial forces can be neglected compared with viscous forces. Thus, a continued propulsion is required to sustain the motion of particles; (ii) Brownian motion on a tiny length scale is a problem when we try to control MNMs precisely; (iii) compared with macroscopic machines, complex micro/nano structures are more difficult and technically challenging to make. To deal with these problems, scientists have exploited many strategies in the past decades. Here we will briefly review some typical ways to design and manipulate MNMs.

In this review, we firstly introduce several different propulsion mechanisms powered by different energy sources. Then we illustrate design strategies of some typical MNMs based on these power sources. Finally, the progress in their applications for water remediation and biomedical microrobots is reported. Different from those reviews in the field which focus mainly on applications of MNMs in specific areas [5,12,13,17], here we focus on the underlying mechanisms for propulsion and the principles for motors’ shape design.

## 2. Methods of Propelling MNMs

It has been widely accepted that an asymmetric field (*Y*) of chemical products or energy is required to actuate particles, which follows a general form:

(1) U=−b∇Y

where *U* is the particle velocity, *b* is the velocity coefficient, and ∇*Y* is the gradient of a potential function *Y* such as pressure, electric potential, solute concentration, or temperature, etc. [20]. Thus, autonomous micromotors can be classified based on the sources used to generate the asymmetric field. In this part, we will overview four common types of micromotors driven by the gradient field generated by chemical sources, light, magnetic field and acoustic waves, respectively.

### 2.1. Chemically Powered Motors

Chemically powered autonomous motion is one of the most widely used mechanisms in the MNMs field. A classic example are the micromotors powered by platinum (Pt)-catalyzed H_2_O_2_ decomposition. In the pioneering work done by Paxton et al. [21], Au/Pt nanowires were reported to exhibit self-propelled capabilities in H_2_O_2_ solutions. This has been followed by many scientific studies due to its simpleness and easy accessibility [19]. In fact, almost any type of microparticle can become a micromotor in H_2_O_2_ solutions if it can be decorated asymmetrically with Pt. Although Pt-H_2_O_2_ system is the dominant approach for propelling micromotors, a variety of other fuels and catalysts have also been discovered for driving the autonomous motion of micromotors [22,23,24,25,26,27,28,29,30,31].

There are three types of mechanisms proposed for understanding the chemically powered autonomous motion, which are self-diffusiophoresis, self-electrophoresis and bubble propulsion. When it comes to phoretic propulsion, three equations are often introduced to depict this phenomenon, including the Navier-Stokes equations:

(2) $$\nabla \cdot u=0\eta \nabla^{2}u=\nabla p+F_{b}$$

where *u* is the bulk velocity, *η* is the fluid viscosity, *p* is the pressure and *F_b_* is the body force; and the species conservation equation:

(3) $$\frac{\partial c_{i}}{\partial t}+\nabla \cdot j_{i}=0$$

where *c_i_* is the concentration of *i* species and *j_i_* is the flux, governed by the generalized Nernst-Planck equation:

(4) $$j_{i}=c_{i}u-D_{i}\left[\nabla c_{i}+\frac{c_{i}}{k_{B}T}\nabla \psi_{i}\right]$$

where *D_i_* is the diffusion coefficient of the solute, *k_B_* is the Boltzmann’s constant, *T* is the temperature and *ψ_i_* is a generalized interaction potential describing the overall interactions between *i* species and environment (for example electrostatic interactions). The three terms on the right side of Equation (4) reflect the advection, diffusion and interactions due to some considerable gradient of *ψ*, respectively [32]. Taking Equations (3) and (4) into Equation (2), theoretical models for specific self-propelled motors can be constructed.

**Figure 1:**
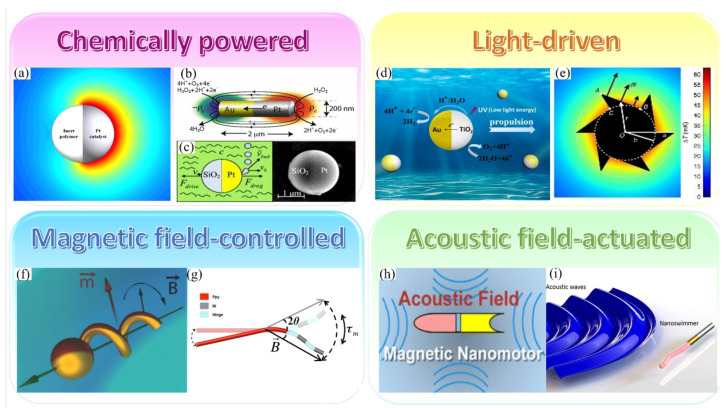
Four common ways to propel MNMs. (**a**) An illustration showing the chemical gradient formed around a Janus microsphere. Reprinted with permission from [33]. (**b**) Pt/Au nanorods swim through a self-electrophoresis mechanism. Reprinted with permission from [48]. (**c**) Microspheres propelled by bubble recoil. Reprinted with permission from [51]. (**d**) An illustration showing motile microspheres driven by photocatalytic reactions. Reprinted with permission from [43]. (**e**) Rotation of a microgear induced by a laser-induced thermal gradient. Reprinted with permission from [60]. (**f**) A schematic graph showing a helical micropropeller moving in a rotational magnetic field. Reprinted with permission from [67]. (**g**) A schematic graph showing a multi-linked nanowire moving in an oscillating magnetic field. Reprinted with permission from [71]. (**h**) An illustration of an acoustic field-powered nanowire with magnetic navigation. Reprinted with permission from [65]. (**i**) A schematic graph showing a nanowire powered by travelling acoustic waves. Reprinted with permission from [75].

In a self-diffusiophoresis locomotion of a particle, a concentration gradient forms near the particle due to asymmetrical reactant/catalyst distribution on it (see one example in Figure 1a) [33]. As different molecules interact with the particle differently, a potential can be defined as $-\nabla \psi \equiv F_{s}$ where $F_{s}$ is the net force experienced by a solute molecule [34]. This force can be integrated and finally converted to a body force represented as $F_{b}=-c\nabla \psi$, which corresponds to the third term of Equation (4) [32]. Different theories and models have been developed to describe the molecular potential *ψ* including Steric forces, van der Waals forces, hydration forces [35] and electrokinetic effects [36,37]. Self-propelled particles based on self-diffusiophoresis were first reported by Howse et al., where half Pt-coated Janus spheres were observed to show directed motion in H_2_O_2_ solutions [38]. This mechanism has been adopted to explain Janus spheres’ autonomous propulsion either in chemical fuels or photocatalytic light irradiation [38,39,40,41,42]. However, the mechanism for the autonomous motion of these Pt-coated Janus particles is still controversial, because it is also found that the low ironic strength of the solution is vital for such propulsion [36,37], which indicates that self-diffusiophoresis is likely not the only driving mechanism for the observed locomotion of these particles. Therefore, Howse et al. later improved their model with an electrokinetic theory, which is in line with the self-electrophoretic mechanism and has been accepted by more and more researchers [43,44,45,46]. In fact, only a few particles based on organic reactions have been demonstrated to swim through self-diffusiophoresis predominantly [22,47], as there were no ions generated during these reactions.

The self-electrophoresis based on an electrical double layer (EDL) model is another mechanism used to explain self-propulsion of particles. When electrochemical reactions occur on the two sides of a particle (for example, a nanorod in Figure 1b) [48], an electric field is generated just like a battery and this forms a charged surface which attracts counterions in the solution. Therefore, electrons flow from anode to cathode while outside ions move inversely, which results in a local current and propels particles in the direction from cathode to anode. Since the properties of EDL are sensitive to the ionic strength of the solution, we can imagine that when salt ions exist in the solution, they would be attracted to the surface of particles (EDL) and thus shield the generated electric field, which then leads to repel reactant ions (mostly protons) and inhibit the reactions. This picture is consistent with the experimental results that the speed of H_2_O_2_-driven particles is reduced dramatically even when trace amount of salt ions exists in solutions [36,37]. A mathematical model can be derived from the Nernst-Planck equation (Equation (4)) [32] with an interaction potential term $\psi_{i}=z_{i}e\phi$, where *z_i_* is the valence of ion species *i*, *e* is the elemental charge and *ϕ* is the electrostatic potential which can be derived using either a flux based model [49] or a kinetic boundary model [50].

Some fuels (e.g., H_2_O_2_, active metal or CaCO_3_) can generate gas phases during reactions, which provide another approach to drive particles. During these reactions, new gas phase seeds appear on particle surfaces, grow up and finally emit into environment, which results in a thrust for particles. This process is known as bubble propulsion. For example, Gibbs et al. have developed a simple bubble propulsion model in which SiO_2_/Pt Janus microspheres can be driven in H_2_O_2_ solutions through O_2_ bubbles detachment, which has also been experimentally confirmed (Figure 1c) [51]. Compared with self-diffusiophoresis and self-electrophoresis, this bubble propulsion mechanism requires a higher reactant concentration to generate bubbles constantly as well as a lower surface tension in the solutions to form bubble seeds easier. But it has advantages in that it is insensitive to salt ions and thus is capable to perform tasks in complicated circumstances. Due to its strong dependence on the particle shape as well as chemical environment, a unified mathematical model for bubble propulsion has not yet been fully solved. But for a specific system consisting of tubular microjets in H_2_O_2_ solutions, models have been developed by either an analytic way [52] or numerical simulations [53].

### 2.2. Light-Driven Motors

Some light has been used to drive MNMs mainly though photocatalytic reactions or photothermal process. When particles asymmetrically coated with photocatalyst materials are put in a certain light field, the photocatalyst will trigger chemical reactions that can result in mass transfer and fluent current around particles, which then lead to the propulsion of particles, in a way similar to that for chemically powered particles. For example, semiconductor TiO_2_ spheres half-coated by noble metals (such as Pt and Au) can absorb light to activate photolysis of water and thus become self-propelled [43,54] (Figure 1d). For photocatalytic reactions to occur, UV lights are often required to afford sufficient energy for electron excitation in materials with high band gaps [43,45,54,55,56]. In recent years, visible-light activated materials such as bismuth oxyiodide [57] has also been developed for such applications.

Besides photocatalysis, the thermal gradient generated by light absorption can also be used to drive particle locomotion. For this purpose, photothermal materials like noble metal and carbon are typically irradiated by light (mostly near infrared (NIR)). This will induce a temperature gradient that can lead to thermophoresis (also termed thermal diffusion or the Soret effect), under which particles can move along the temperature gradient direction. The propulsive velocity *U* can be written as $U=-DS_{T}\nabla T$, where *S_T_* is the Soret coefficient [58]. For a silica/Au Janus sphere with a radius of *R* driven by thermophoresis in a typical laser beam, the particle velocity can be written as:

(5) $$U=-DS_{T}\frac{\Delta T}{3R}$$

where *S_T_* of a Janus sphere is interpreted as the average of the two coefficients of the bare half (i.e., none-coated half) and the other Au-coated half of the Janus sphere, ∆*T* is the temperature difference between the two halves of the Janus sphere [59]. Interestingly, under appropriate conditions, the generated thermal gradient due to light absorption can also induce thermocapillary forces to propel particles. For example, Maggi et al. showed that by putting asymmetric micron gears at an air-liquid interface and under light illumination, the generated thermal gradient due to the light absorption of gears induced variations in the local surface tension of the air-liquid interface, which then generated a capillary torque and drove gears’ rotations (Figure 1e) [60]. This Marangoni type propulsion is 10^5^ stronger than that from thermophoresis in the same temperature gradient [60].

### 2.3. Magnetic Field-Controlled Motors

Magnetic fields are easier to use and are unscreened even in electrolyte solutions compared with electric fields, thus it has been attractive to develop magnetic field-driven micromotors. For a ferromagnetic particle, when it is in an inhomogeneous field, the magnetic force can be written as:

(6) $$\vec{F}=\frac{1}{\mu_{0}}\left(\vec{m}\cdot \vec{\nabla }\right)\vec{B}$$

where *μ*_0_ is the magnetic permeability of vacuum, $\vec{m}$ is the permanent magnetic moment and $\vec{B}$ is the magnetic flux density. We note that this equation is not suitable if the magnetic field is stronger than the coercive field of the material [61]. According to Equation (6), we can see that a gradient of magnetic field is required to drive the motion of ferromagnetic particles. And the driving force scales linearly with both the magnetic dipole moment and the field gradient. Using an inhomogeneous field, Liu et al. reported that Ni-Au nanorods can swim in several µm⋅s^−1^ under a T⋅m^−1^ magnitude field gradient [62]. A homogeneous static magnetic field, however, cannot directly propel motors but can be used to control their alignment and steer the direction of their motion. Thus, adding ferromagnets to micromotors and exerting a homogeneous magnetic field have become a regular method for particle navigation [63,64,65].

Since a magnetic dipole would tend to align with the field direction to minimize the free energy when it is put in an external magnetic field, this principle can be utilized to drive the rotation of ferromagnetic particles by using a rotating magnetic field [61]. Inspired by biological micro swimmers, such rotational motion can be converted into translational motion by attaching ferromagnets onto flagellum-like structures (e.g., helix [66,67] (Figure 1f), nanowires [68] or bacterial flagella [69]).

Oscillating magnetic fields can also be used to drive particle motion. However, in order to make a net translational displacement, time-symmetric motion has to be broken as reciprocal motion does not result in net displacement which is also known as scallop theorem first presented by Purcell [70]. For example, Jang et al. reported a type of three-link nanoswimmers consisting of one polymeric and two magnetic metallic nanowires linked by hinges (Figure 1g) [71]. The strategy for this simplest structure to break a time-reversible motion is mainly through the undulation of its flexible tail. Under an oscillating magnetic field, this swimmer can swim forward in a maximum speed of 0.93 body-lengths s^–1^.

### 2.4. Acoustic Field-Actuated Motors

Recently, acoustic field-actuated MNMs have drawn a lot of attention due to their great potential in drug delivery applications. These micromotors such as concave nanowires (Figure 1h) and nanoshells [65,72,73] typically have asymmetric structures. When such particles with asymmetric geometry absorb, scatter or reflect sound in an acoustic field, different acoustic radiation forces are exerted to the particles which can result in a pressure gradient to propel them directionally.

Theoretically, for a micromotor suspension in an acoustic field, the acoustic radiation force F on a micromotor can be expressed as the following [14,74]:

(7) $$F=-\left(V\left(t\right)\nabla p\left(r,t\right)\right)=-\left(\frac{\pi p_{0}^{2}V\beta_{w}}{2\lambda }\right)\Phi \left(\beta ,\rho \right)\sin\left(2kd\right)$$

where *k* is the wave number, *λ* is the wavelength, *V* is the volume of the particle, *p*_0_ is the pressure amplitude and *d* is the distance between the particle and the node or antinode. Φ(β,ρ) is given by the following equation:

(8) $$\Phi \left(\beta ,\rho \right)=\frac{s-\rho_{c}-2\rho_{w}}{2\rho_{c}+\rho_{w}}-\frac{\beta_{c}}{\beta_{w}}$$

where $\rho_{c}$ and $\rho_{w}$ are the density, and $\beta_{c}$ and $\beta_{w}$ are the compressibility of the particle and the solution, respectively. From Equation (7), we can see that the acoustic radiation force decreases dramatically when the particle size decreases, so particles with far smaller size than the acoustic wavelength can be hardly controlled by the acoustic field.

For biological applications, those micromotors driven by acoustic standing-waves are typically low efficient due to the complex biological environment (for example, in a human body that have complicated boundary conditions) and thus affect the formation of standing waves. Hence, motors driven by acoustic travelling waves have also been explored. By connecting a flexible polypyrrole tail with a bimetallic (Ni/Au) head, Ahmed et al. fabricated a different type of nanoswimmer that can swim in a travelling acoustic wave. The flagellum-like tail of this nanoswimmer will oscillate under acoustic excitation, and thus generate a propulsion force for its swimming (Figure 1i) [75].

## 3. Strategies in Designing MNMs

In the past decades, the field of active matter has thrived and exploited many imaginative strategies to actuate particles. In general, these strategies fall into two categories. The first strategy is to apply an external force on particles by adding an asymmetric field gradient. The second one is to design asymmetric particles, which can convert energy into mechanical work even in a symmetric external field, as these particles can themselves form a local asymmetric field when they absorb energies and thus propel themselves. In this section, we will mainly discuss the second strategy.

### 3.1. Janus Spheres

Janus spheres are micro/nanospheres with half surfaces coated by functional materials, and thus have asymmetric physical and/or chemical properties on two sides. When they are put in a symmetric field like chemical reactants or light, they can themselves generate a gradient field and become autonomous motive. A variety of Janus spheres driven by different propulsion mechanisms have been developed.

**Figure 2:**
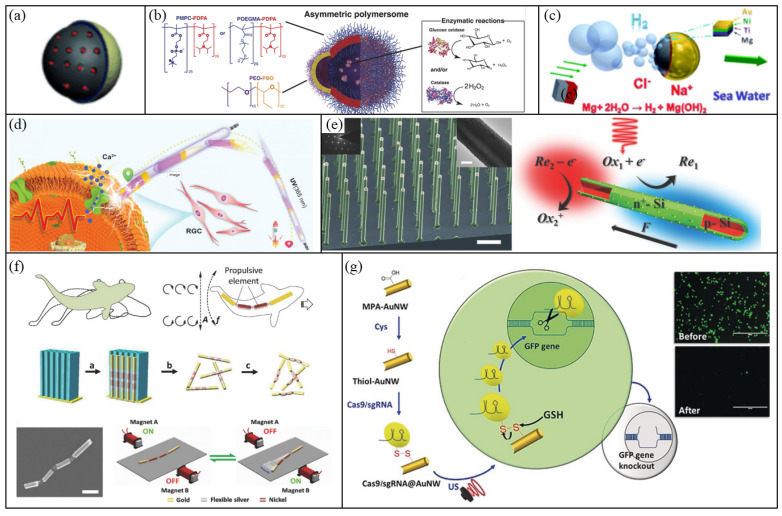
Examples of Janus spheres and nanowires designed for MNMs. (**a**) Self-propelled Janus mesoporous silica nanomotors for drug delivery. Reprinted with permission from [76]. (**b**) Schematic representation of an asymmetric chemotactic vesicle (polymersome). Reprinted with permission from [86]. (**c**) An illustration of a magnetic field-guided microsphere swimming in sea water through bubble recoil. Reprinted with permission from [29]. (**d**) An illustration showing TiO_2_-Au nanowires used for subretinal repair. Reprinted with permission from [100]. (**e**) Spectrally tunable light-driven silicon nanowires. Reprinted with permission from [101]. (**f**) Magnetic field-powered flexible nanowires under an oscillating magnetic field. Reprinted with permission from [103]. (**g**) A schematic graph showing acoustic field-actuated nanowires used for genetic editing. Reprinted with permission from [110].

Chemically powered Janus spheres are one type of widely used micromotors. A classic example is the Pt/polystyrene Janus spheres powered by H_2_O_2_ decomposition [38]. For the Pt/polystyrene Janus spheres, polymer microspheres mainly serve as cores to sustain the particle geometry without additional functions. To improve the efficiency of space usage, porous materials such as mesoporous silica or activated carbon core have been used to replace the solid polymer core in these Janus spheres (Figure 2a) [76,77,78,79]. The modified porous Janus micromotors can be loaded with cargos, which are more suitable for applications such as drug delivery. The Pt layer coated on a Janus sphere is typically fabricated by physical vapor deposition, which would lead to a smooth deposition surface. However, such smooth surface is difficult to form bubble seeds, and leave self-diffusiophoresis and/or self-electrophoresis to be the main propulsion mechanisms for these Janus spheres [80]. By contrast, when a Pt layer with a rough surface is used, the motion of Janus spheres will also benefit from the bubble propulsion, and this leads to a multifold increase in particle speed and an enhanced tolerance to ionic conditions [80,81,82,83]. Apart from the Pt-H_2_O_2_ system, a variety of other catalyst-reactant systems have also been developed such as Pd-H_2_O_2_ [24], Ag-H_2_O_2_ [25], TiO_2_-H_2_O_2_ [84]_,_ Grubbs’ ring-opening metathesis polymerization catalyst-norbornene [47], iridium-N_2_H_4_ [26] and urease-urea [85], etc. Recently, a new type of Janus vesicle particles (Figure 2b) have been fabricated by Joseph et al. [86]. Unlike traditional Pt/polymeric core Janus spheres, where the Pt layer is on part of the outside surface of a particle, for these vesicle particles, catalysts (for example, glucose oxidase and catalase) are in the inner space of particles enclosed by an asymmetric polymer shell. Due to different properties on the two sides of the shell, mass transfer is asymmetric across vesicle particles and thus leads to their self-propulsion. These vesicle particles also exhibit chemotactic behavior in glucose gradient and can be employed to cross blood-brain barrier in target therapy [86].

All the above studies focus on the Janus particles consisting of catalyst materials. However, Janus particles can also be made of fuel materials. For the latter case, spheres of a solid fuel (mostly active metals) are coated asymmetrically by inert materials. Then, when these Janus spheres are put in reactant solutions, only the exposed part of fuel surfaces (i.e, the part that is not covered by inert materials) will react with solutions. Consequently, particles experience a net thrust, for example through bubble propulsion, along the direction from the exposed side toward the covered side and become motile (see one example in Figure 2c). Many different fuel-reactant systems have been employed for this type of Janus particles including Mg-water [29,87,88,89], Zn-water [90], Al-water [23,28], gallium-water [28], CaCO_3_-acid [91] and poly (2-ethyl cyanoacrylate)-water [92] etc. Such micromotors have good tolerance to ionic conditions and thus work in a broad range of solutions. However, one apparent drawback is that they are one-time-use micromotors and will break down after their fuel cores have been exhausted.

Besides chemical fuels, other power sources like light have also been applied to drive Janus particles. For example, microspheres decorated with Au caps can absorb NIR, which result in a temperature increase locally, so they can move by thermophoresis [93,94,95]. Interestingly, a locally high temperature induced in such Janus spheres can also be utilized as thermal therapy for tumors [93]. Another common type of light-driven micromotors are those Janus particles decorated with photocatalytic materials like TiO_2_ [43,54]. A solid TiO_2_ microsphere with half coated by noble metals can absorb UV light and boost photochemical reactions which degrade water into H_2_ and O_2_. Constrained by the reaction rate and convex geometry shape, these motors are generally propelled by self-electrophoresis rather than bubble recoil.

### 3.2. Nanowires

Nanowires are another type of MNMs designed by their asymmetric properties on the two sides of rods. Therefore, some bimetallic microrods are also known as Janus rods. They are typically several micrometers in length and hundreds of nanometers in diameter. Like Janus spheres, nanowires can be controlled by multiple approaches while nanowires have also their unique manipulation strategies due to their tiny size and structural flexibility.

The first nanowire motor was reported by Paxton et al. in 2004 [21], where they fabricated Au-Pt bimetallic nanorods and found them self-propelling in H_2_O_2_ solutions. They claimed that the decomposition of H_2_O_2_ on Pt side induced a gradient around nanorods in both O_2_ concentration and temperature, and led to a difference of interfacial tension between two sides, which is responsible for the locomotion of nanorods. However, this mechanism can’t explain why catalase-loaded poly(pyrrole)−Au nanorods showed no observed axial movement in Mallouk’s experiment [96]. This implies that metals or other conductors are necessary for bimetallic nanorods and self-electrophoresis mechanism (illustrated in Figure 1b) was proposed for their autonomous motion in H_2_O_2_ solutions. Besides hydrogen peroxide, other fuels such as I_2_ and Br_2_ [30] have also been reported. Although there are studies reported about the usage of bimetallic nanowires in cargo delivery [97,98], the low working power and incompatibility with high ionic conditions have limited their further applications. The working power can be improved by mixing carbon nanotubes into Pt side which can lead to a speed increase by over ten-fold [99], whereas the incompatibility with high ionic conditions is a consequence of self-electrophoresis propulsion mechanism, so to circumvent this problem, nanowires with different propulsion mechanisms have been developed. For example, Chen et al. reported a type of ultraviolet light-driven TiO_2_-Au Janus nanorod [100]. Due to its good biocompatibility, precisely wireless control and inherent photoelectricity, this type of motor has been applied in optoelectronics for subretinal repair (Figure 2d). Using doped silicon materials, Wang et al. also fabricated light-driven nanowire motors (Figure 2e) [101]. After adding two-electron/hole scavenger couple (H_2_O_2_ or hydroquinone) in water as a redox shuttle, these nanowires can move in water with a swimming speed of 9.6 µm s^−1^ under a light intensity of 3 mW cm^−2^. Interestingly, the spectral response of these doped silicon nanomotors can be adjusted from visible to NIR light by controlling their synthetic diameters.

Similarly, ferromagnetic nanowire motors driven by a magnetic field have also been explored. Among them, inspired by natural microswimmers, many are designed to have flexibility in their structures, so that they can harness magnetic energy into translational motion in an oscillating or rotating magnetic field [68,71,102,103,104,105,106]. For example, Li et al. fabricated gold and nickel nanorods linked by flexible porous silver hinges. Then under an oscillating magnetic field, these fish-like nanowires can swim with a speed of 30 µm s^−1^, which were driven through the propulsive nickel segments that can bend the nanowires periodically and thus generate travelling wave motions (Figure 2f) [103]). For rigid and non-chiral nanorods, a nonuniform magnetic field (i.e., the gradient of a magnetic field) can be used to generate their translational motion [62]. Interestingly, by taking advantage of the boundary effect that typically increases the hydrodynamic drag on particles when they move near a solid wall, Zhang et al. realized the translational propulsion of a rigid and nonchiral nickel nanowire through its tumbling motion induced in a rotating magnetic field [107].

Besides the aforementioned chemical fuels, light and magnetic field powered nanowires, acoustic waves have also been used to drive nanowire motion. For such acoustic motors, asymmetric concave structures in them are often required which can scatter ultrasound waves differently and thus result in a pressure gradient to propel them [72,108,109]. Ultrasound propelled nanowires were firstly demonstrated by Wang et al. [72]. After that, they have shown their great potential in biomedical applications [4,5,110,111,112] due to their unique properties such as tiny size and ultrasound’s sufficient force, good biocompatibility and sensitive control etc. For example, by loading nanowires with Cas9/sgRNA molecules, Hansen-Bruhn et al. showed that these nanowire motors rapidly internalized into GFP-expressing B16F10 cells under ultrasound, which then cleaved the targeted GFP genomic sequence specifically. This resulted in an increased knockout efficiency of 80% within 2h of cell incubation, compared with about 30% when their static counterparts were used under the same conditions. Moreover, with these nanowire motors, as little as 0.6 nM of Cas9/sgRNA molecules were needed due to their high knockout efficiency (Figure 2g) [110]. Similarly, many other functional biomacromolecules can also be loaded onto this type of intracellular shuttle to perform different tasks [4,111,112], which have enriched the operation methods in both cell biology and molecular biology.

### 3.3. Microjets

**Figure 3:**
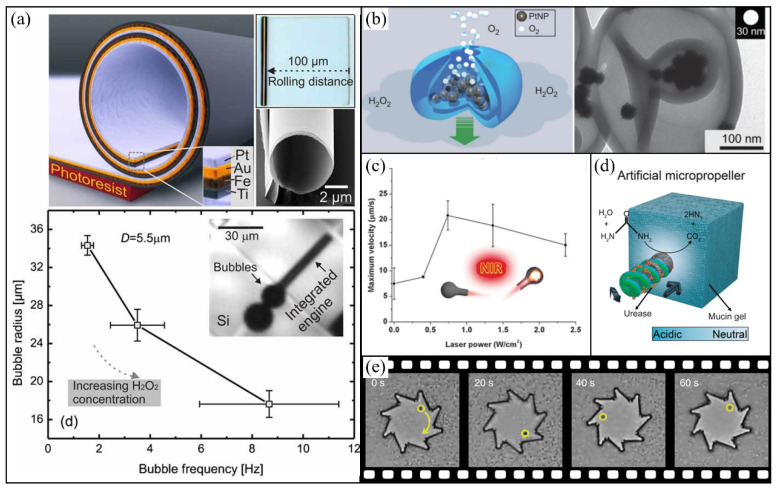
Examples of microjets and chiral motors. (**a**) Microtubular motors fabricated by a rolling-up technology. Reprinted with permission from [113]. (**b**) An illustration of a polymer stomatocyte and its transmission electron microscope image. Reprinted with permission from [119]. (**c**) Carbonaceous nanobottles’ velocity as a function of light intensity. Reprinted with permission from [123]. (**d**) Magnetic powered micropropellers decorated with urease can drill into mucin gel. Reprinted with permission from [128]. (**e**) Bacteria-driven rotation of microgears. Reprinted with permission from [130].

Microjets are microswimmers propelled by recoil forces due to the ejection of either bubbles or liquid. They have wide applications due to their powerful thrust and robustness in high ionic media. A typical example of microjet is strain-engineered microtubes shown by Solovev et al. The microtubes were made by rolling up a Ti/Fe/Au/Pt multilayer, in which the Pt inner layer is responsible for decomposing H_2_O_2_ fuel in solutions to provide propulsion while the Fe layer can respond to an external magnetic field for steering the particle orientation (Figure 3a) [113]. When these microtubes were put in H_2_O_2_ solutions, they showed self-propelled motion with a speed of up to 2 mm/s.

The propulsion process of such microtubular jets generally includes three steps: Firstly, the fuel solution wets the catalytic material containing energetically favorable nucleation points, where O_2_ (or other gas) accumulates and grows as bubbles. Then, bubbles migrate towards larger opening of a microtube. And finally, the bubbles emit out of the tube and provide a powerful thrust. Meanwhile, fresh fuel is sucked into the tube through the other opening from surrounding solutions to start a new circulation [19]. Theoretical models have been developed to provide guidance in designing efficient microtubes with optimized parameters including length, radius [114] and semi-cone [115] of microtubes, as well as surfactant concentration [116]. Microtubes with other power sources such as Zn-acid [27], TiO_2_-UV light [117], and perfluorocarbon-ultrasound [118] have also been reported for different applications.

Besides microtubes, other shaped microjets have also been developed. For example, Wilson et al. fabricated bowl-shaped microjets which are polymer vesicles with platinum nanoparticles inside (Figure 3b) [119]. For such a bowl-shaped microjet, H_2_O_2_ fuel can diffuse freely into polymersomes and are decomposed by Pt, then bubbles emit from the opening of the microjet, which provides it a propulsion force, in a way similar to the propulsion of monopropellant rocket engine. Compared with chemically powered Janus particles, the directionality of the movement of these bowl-shaped motors is easier to be controlled via their openings. By appropriate surface and/or core modifications, these bowl-shaped microjets can be used for different applications [120,121,122].

Recently, as an extension of similar opening-involved structures, a new type of microjet with a different shape, carbonaceous nanobottle, has been reported [123]. Carbonaceous nanobottles absorb cyclic NIR laser and heat the internal fluid rapidly, which causes propulsion through the ejection of the heated fluid periodically from their open neck (Figure 3c). Thus, the working propellant of these light-driven nanobottles is liquid, rather than bubbles as in the aforementioned microjets. But this type of structure can also adapt chemical fuels by encapsulating Pt nanoparticles into carbonaceous nanoflasks [124] or silica-based nanobottles [125,126], which exhibit promising applications in drug delivery and release etc.

### 3.4. Particles with Chiral Structures

Beyond the aforementioned micromotors that perform translational motion through breaking a mirror symmetry to generate the field gradient required for propulsion, micromotors with rotational motions have also been developed. These particles typically contain chiral structures.

A classic example is a bacterial flagellum, which powers the bacterial swimming motion through the rotation of helical filaments [127]. Inspired by it, Zhang et al. fabricated artificial bacterial flagella (ABF) through a self-scrolling mechanism due to different stresses in InGaAs/GaAs/Cr trilayer pattern [66]. Then by connecting these ABF with thin soft-magnetic heads, the obtained particles showed their translational motion through the rotation of ABF induced in a rotational magnetic field [66]. By immobilizing urease on particle surfaces, Walker et al. developed enzymatically active biomimetic micropropellers (Figure 3d), which could not only be actuated by rotational magnetic fields but also change the rheological properties of the viscoelastic biological media. Consequently, these micropropellers can get through mucus barriers [128]. While the majority of reported helical micromotors is propelled by magnetic fields, Shang et al. also showed a hydrogen peroxide-driven helical micromotor formed by dispersing Pt nanoparticles into a helical gel [129]. This chemical powered helical motor can reach a maximum speed of 0.6 µm s^−1^ by bubble recoil.

To harness the motion of bacteria to power micromotors has been a fascinating goal in nanotechnology. As a proof of concept, by employing microgears which have asymmetric teeth in an active bacteria bath, Leonardo et al. showed these microgears can rotate unidirectionally driven by the self-assembly of motile *Escherichia coli* cells along the rotor boundaries [130] (Figure 3e). The asymmetric geometry of microgear is important as it breaks the symmetry necessary for the rectified motion of particles [130,131]. For such bacteria-powered microgears, the concentration of bacterial cells is critical as it should be high enough so that bacterial collective motion can emerge, but should also be below a threshold value after which microgears will stop rotating likely due to the onset of quorum sensing and biofilm formation [131]. Microgears with other propulsion strategies like the photothermal effect [60] and electrokinetic effect [132] have also been reported in recent years.

### 3.5. Other 3D Micromotors with Nontraditional Shapes

With the advances in fabrication technology such as direct laser writing (DLW) [133] and origami folding techniques [134], 3D micromotors with nontraditional shapes beyond the classical shapes of the micromotors introduced above have also been developed. These micromotors can exhibit impressive fascinating functions through well-designed complex structures. For example, Zeng et al. fabricated light driven microscopic walkers by DLW [135], which consist of a main body of liquid crystalline elastomer and four legs of acrylic resin. These walkers can walk and jump through the contract-expand cycles of the main body under chopped 532 nm laser excitation at certain frequencies. By appropriately designing particles with internal body cavities which can trap bubbles, bubble-based microswimmers driven by ultrasound have also been reported, which can move with a much faster speed (up to mm s^−1^) than other common synthetic swimmers in the same size range [136,137,138].

Recent advances in integrating artificial intelligence systems into micromotors further expanded their functions. For instance, Miskin et al. demonstrated walking microscopic robots with photovoltaics body and legs that can bend in response to electrochemically driven adsorption [139]. Notably, these microscopic robots are compatible with standard silicon electronics, providing them the capability to interact with their microenvironment using local sensory input and feedback. Cui et al. constructed a microscale ‘bird’ which consists of encoded panels linked by hinge springs and can be programmed by an external magnetic field [11]. By polarizing nanomagnets on panels with magnetizing field sequences to store the shape-morphing information, this micromachine exhibited rich behaviours including ‘flapping’, ‘hovering’, ‘turning’ and ‘side-slipping’. Similarly, other 3D micromotors incorporating compound structures such as Archimedes screw-pumps [140] or 3D-printed structural colors have also been illustrated [141] and show great potential for applications.

## 4. Applications of MNMs

Compared with passive colloidal particles, active micromotors have drastic movement which leads to convection around motors in micro scale and/or high diffusion rates in macro scale. These unique properties make them an attractive candidate for a variety of applications ranging from environmental sensing, water remediation to biomedical applications such as drug delivery and microsurgeries etc. Here we use micromotors in water remediations and biomedical robots as examples to illustrate their applications.

### 4.1. Water Remediation

**Figure 4:**
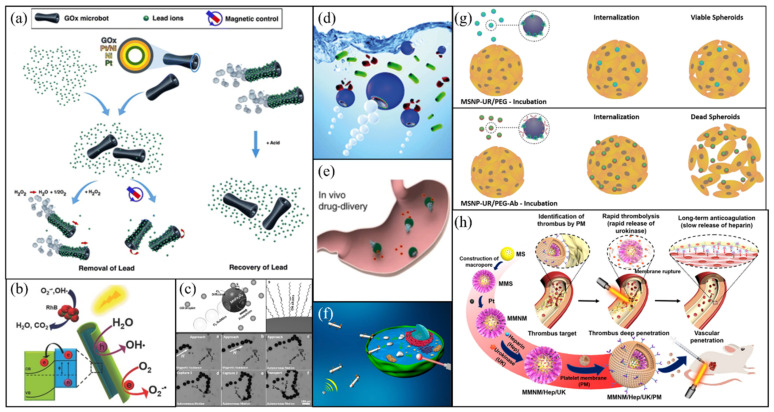
Applications of MNMs in water remediation and biomedical microrobots. (**a**) Functionalized microtubes remove Pb^2+^ in water and then are recycled with magnets. Reprinted with permission from [142]. (**b**) Scheme showing photocatalytic degradation of organic molecules using light-driven microtubes. Reprinted with permission from [143]. (**c**) MnFe_2_O_4_ micromotors can remove oil pollutants directly from contaminated water by physical adsorption. Reprinted with permission from [145]. (**d**) A schematic drawing of chitosan coated Janus spheres in cleaning bacteria contaminated water. Reprinted with permission from [146]. (**e**) An illustration displaying antibiotic modified Janus sphere to cure stomach infections. Reprinted with permission from [150]. (**f**) An illustration showing fusible acoustic driven Ga nanowires to kill a HeLa cell. Reprinted with permission from [152]. (**g**) Comparison between micromotors with and without antibody in the internalization efficiency for 3D spheroids of bladder cancer cells. Reprinted with permission from [154]. (**h**) Schematic illustration of the components of platelet-derived porous nanomotor and their functions in thrombolytic treatment. Reprinted with permission from [79].

Micromotors, due to their active movement, can be smoothly and thoroughly transferrable with water and thus have been increasingly applied in water remediation. For example, to purify water from Pb^2+^ contamination, Vilela et al. developed an integrated microtubular system, which included a power system (inner Pt layer) to trigger bubble propulsion, a navigation or recycle system (middle Ni layer) for magnetic remote control and a purification system (outer graphene oxide layer) for Pb^2+^ adsorption (Figure 4a) [142]. With this system, over 80% of Pb^2+^ was adsorbed in 1 h, far more efficient than the traditional adsorbent.

Organic pollutants are another common contamination source of water. By combining the photocatalytic properties of TiO_2_ with the very high specific surface area of tubular shape, Mushtaq et al. fabricated coaxial hybrid TiO_2_-PtPd-Ni nanotubes (Figure 4b) which showed excellent photocatalytic activities to degrade organic pollutants not only under UV light but also under visible light and natural sunlight [143]. They showed that using these nanotube motors, organic pollutant rhodamine B could be degraded 100% in 30 min under natural sunlight and in 50 min under visible light. The multicomponent design of nanotubes provides multiple ways including magnetic field, acoustic field and chemical fuels to power and control their autonomous motion, which not only improves the efficiency of pollutant degradation but also enables them to work properly under different environments.

Similarly, recent work reported by Kochergin et al. describes photocatalytic micromotors consisting semiconducting sulfur- and nitrogen-containing donor-acceptor polymer [144]. Under visible light, these micromotors are motile via a self-diffusiophoresis mechanism, during which radical species such as ·OH and ·O^−^ are produced by photocatalytic reactions with water and oxygen. Meanwhile, these radical species can also react with organic dyes and result in an apparent rate constant of 0.039/min for the rhodamine B degradation reaction, comparable to some of the benchmark photocatalysts [144]. Compared with TiO_2_-based or other inorganic light-driven micromotors which typically contain costly heavy and precious metals, these polymer micromotors are based exclusively on an organic polymer framework, so they are economically cheap and their optical properties can also be precisely engineered using synthetic approaches or through post-synthetic modifications. All these merits have made them an attractive candidate for a wide range of applications including water purification and environmental sensors, etc.

The aforementioned micromotors designed for water purification are typically multilayered. On one hand, this type of design enables them to have possible multiple functions and to response to multiple stimuli signals. On the other hand, it often requires a complicated fabrication process, which may limit their practical applications. So, finding alternative ways to make functional micromotors with simple structure and easy fabrication process is still urgently demand. Toward this goal, Mou et al. fabricated MnFe_2_O_4_ micromotors which are single-layered pot-like MnFe_2_O_4_ hollow microspheres with an opening in the shell through a facile, large-scale fabrication process [145]. These micromotors are propelled by ejecting bubbles from the opening, resulted from the preferentially growth of bubbles on the inner concave surfaces. More importantly, it was demonstrated that they can remove oil pollutants directly from contaminated water by physical adsorption (Figure 4c), as their outer surfaces are hydrophobic due to the oleic acid molecules on the surfaces remained from the synthesis process.

With appropriate modifications, micromotors can also be used to kill pathogenic microorganisms in water. For instance, by coating water-powered Mg-based micromotors with antibacterial biopolymer chitosan, Delezuk et al. showed that such active micromotors displayed a 96% bacterial-killing efficiency within 10mins, about 27 times higher than that when static chitosan-coated microparticles were used (Figure 4d) [146]. Moreover, since these micromotors are driven through a bubble propulsion mechanism, they perform well in seawater treatment. Similar micromotors equipped with other antibacterial materials such as silver [88,147], lectin [148], or lysozyme [149] have also been reported.

### 4.2. Biomedical Microrobots

The motion of MNMs can be precisely controlled through for example a magnetic field, so MNMs can be guided to the target site. Under proper stimuli conditions, they could cross certain biological barriers and penetrate deeper in tissues. Moreover, through synthetic process or post-synthesis surface modifications, they can be made to be biocompatible and/or biodegradable. All these merits make micromotors (also called microrobots) suitable for biomedical applications in, for example, targeted drug delivery, diagnosis, bacterial infection treatment and cancer therapies etc., and have attracted significant interests in recent years [6].

de Ávila et al. reported the first in vivo Mg-based therapeutic micromotors to treat gastric *Helicobacter pylori* infections in a mouse model. The micromotors consist of a Mg core coated by a clarithromycin (an antibiotic)-loaded poly\(lactic-co-glycolic acid) layer which is then covered by an outside chitosan layer. When these micromotors were put in a gastric acidic environment, they were propelled by the reaction of magnesium with gastric acid. Then when they reached the stomach wall, the chitosan layer helped them to adhere to the wall and resulted in an effective local release of clarithromycin against *H. pylori* infection (Figure 4e) [150]. Compared with passive drug carriers, these active Mg-based micromotors displayed a significant bacteria burden reduction. Moreover, beyond the propulsion, the reaction of magnesium with gastric acid also depleted protons quickly, thus no need to use proton pump inhibitors as in the case of free drugs which can lead to adverse effects if in long term use.

Biological environments vary in different tissues/organs. To cope with applications in such different environments, beyond Mg-based micromotors, other biocompatible fuels powered, particularly, enzyme catalysis powered micromotors have also been explored. By coating urease onto mesoporous silica-based core-shell particles, Hortelão et al. fabricated urease-powered nanobots for targeted drug delivery, which showed enhanced anticancer efficiency for Hela cells resulted from the synergistic effect of the drug release kinetics’ enhancement and ammonia produced from catalytic decomposition of urea [151]. Similar urease-driven polymer nanomotors have also been developed by Choi et al. [78], which showed great potential in drug delivery applications for treating bladder related diseases.

For biomedical applications, directional control of micromotors is important, particularly for performing tasks that are target-specific. One way to realize the directional control for external-field responsive micromotors is through external fields. For example, Wang et al. fabricated an oxide film coated liquid Ga nanorod, which can be propelled and navigated by an acoustic field (Figure 4f) [152]. They showed that these acoustic driven nanorods could actively seek HeLa cells and when these motors drilled into cells, they transformed into droplets due to the removal of the oxide layer of nanorods in the acidic endosomes, then the HeLa cells could be killed through photothermal effect of the liquid Ga droplets under illumination of near-infrared light. Similarly, micromotors of FeGa@P(VDF-TrFE) core–shell magnetoelectric nanowires have also been shown to be able to perform targeted drug delivery under external magnetic field-control [153].

Beyond the external-field guided micromotors, another common way to realize target-specific tasks is to modify micromotors with appropriate target-specific binding agents such as antibodies. For instance, by covalent binding anti-FGFR3 antibody, Hortelão et al. fabricated urease-powered mesoporous silica micromotors, which specifically target bladder cancer cells. They showed that these antibody-modified active micromotors exhibited four-fold higher internalization efficiency for 3D spheroids of bladder cancer cells compared with active micromotors without the antibody (Figure 4g) [154].

A recent work done by Wan et al. showed an improvement on target-specific micromotors by combining the advantages of both external-field manipulation and target-specific binding agents, together with a proper structural design of particles [79]. The micromotor consists of platelet membrane (PM) coated mesoporous/macroporous silica particles with platinum (Pt) nanoparticles distributed among macroporous structures (Figure 4h). They are propelled through Pt-induced thermophoresis under NIR irradiation. The PM layer would help these micromotors to aggregate at thrombus sites, then under NIR illumination, these micromotors become active motile and thus can penetrate deeper into the thrombus where two drugs, thrombolytic urokinase (UK) loaded in macroporous to recanalize veins and heparin (Hep) loaded in microporous to prevent thrombosis regeneration can be released in a regulated manner through NIR irradiation. Therefore, these micromotors can both clean the clots and inhibit their regeneration, which has been confirmed in both static/dynamic thrombus and rat model [79].

## 5. Conclusions

In summary, we presented an integral profile of MNMs progress in recent years. Four kinds of power sources to actuate MNMs were discussed, which are chemical fuels, light, magnetic and acoustic field. Typical propulsion mechanisms of each power sources have been introduced to provide a guidance in designing high-performance MNMs with different geometries. Some impressive MNMs for environmental remediation and biomedical applications have also been illustrated, all of which rely on their enhanced motilities supplied by basic MNMs.

As the four kinds of power sources have different requirements for driving micromotors, when it comes to specific applications, some power sources will be more appropriate than others. Generally speaking, when designing environmental cleaners, chemical and light powered micromotors are preferred because they can react with substances in solutions or absorb sunlight to propel themselves without adding extra energy sources [2,17]. Ferromagnetic materials on these particles can be used only for recycle, rather than for propulsion by magnetic fields. When designing for drug delivery, micromotors driven by each of the four kinds of power sources have been reported, but at the current stage it seems that those actuated by external fields show a better performance partly because of their better controllability as shown in small animal models like mice [6]. However, since external fields would typically decay when body gets larger, whether this is still the case in large human bodies is unclear. Alternatively, Schmidt et al. suggested that targeted therapy can be achieved by multi-energy propulsion with two steps [13]. At the first step, magnetic field is used to navigate micromotors to gather them near lesions which is called long-range targeting. Then, short-range targeting towards individual cells is realized by micromotors’ chemotaxis and penetration. Constrained by biocompatibility, only micromotors propelled by mild reactions (e.g., enzymatic reaction) would be appropriate for such in vivo biomedical applications. In this sense, acoustic micromotors might be a good candidate to use for these intracellular operations due to their high penetration and good biocompatibility, which have been demonstrated recently by Venugopalan et al. [5].

Beyond the traditional synthetic micromotors, another type of MNMs—biohybrid motors constructed by the integration of microorganisms and synthetic components (often made of soft materials), has also been explored. In a biohybrid motor, the microorganism part acts as a biological power source to propel the motor under physiologically compatible conditions, while the synthetic component part provides the designed specific functionalities like drug releasing or bioimaging [155]. Thus, biohybrid motors combine the advantages of excellent inherent biocompatibility and taxis from microorganisms and a diversity of available functionalities from synthetic components. With all these advantages, biohybrid motors have grown to be an extremely promising category of microrobots in recent years and significant progresses in biomedical applications have been made [155,156,157]. But since this review mainly focuses on synthetic MNMs, such biohybrid motors are not included. Interested readers are referred to recent reviews [155,156,158].

Although a magnificent progress has been made, the development of the MNMs field is just at the beginning and there are many important outstanding questions that remain to be understood in order for their further applications. One of the substantial challenges in the MNMs field is how to design intelligent microrobots based on self-propelled micro/nanoparticles. Various proof-of-concepts have been demonstrated from different perspectives including material science [9], microbiology [158] and microelectronics [139]. But how robust the performance of these microrobots is under varied environments requires further exploration.

More challenges come when MNMs are used in real applications. For example, the energy conversion rate of these motors needs to be greatly improved, so that the requirement for their working conditions could be less strict while their working duration time can be largely expanded. Moreover, compared with in vitro experiments, rheology, boundary conditions and chemical compositions of fluid all will change dramatically when MNMs are used in vivo applications [12]. Because of this, for biomedical applications such as targeted therapy and microscale surgery, developing efficient and precisely controlled micromotors in complex media is especially prioritized. In addition, for such biomedical applications, biocompatibility and biodegradability are also important problems that need to be considered when choosing materials for MNMs. For environmental remediation, which usually requires a large amount of cleaner, manufacture cost should also be reduced to promote the usage of MNMs.

While the design and manipulation techniques for MNMs gradually mature, to what extent they can be used in real applications relies on an interdisciplinary effort, for example, how to choose a proper actuating method for a specific application circumstance and/or integrate functional components on MNMs for a specific target, etc. In this sense, this field especially welcomes researchers from different disciplines with different backgrounds to work together to build a magnificent “micro world”.

## Data Availability

The data in the paper is in line with MDPI Research Data Policies.

## References

[1] Saper G., Hess H. (2020). Synthetic Systems Powered by Biological Molecular Motors. Chem. Rev..

[2] Parmar J., Vilela D., Villa K., Wang J., Sánchez S. (2018). Micro-and Nanomotors as Active Environmental Microcleaners and Sensors. J. Am. Chem. Soc..

[3] Guix M., Orozco J., García M., Gao W., Sattayasamitsathit S., Merkoçi A., Escarpa A., Wang J. (2012). Superhydrophobic Alkanethiol-Coated Microsubmarines for Effective Removal of Oil. ACS Nano.

[4] De Ávila B.E.-F., Angell C., Soto F., Lopez-Ramirez M.A., Báez D.F., Xie S., Wang J., Chen Y. (2016). Acoustically Propelled Nanomotors for Intracellular siRNA Delivery. ACS Nano.

[5] Venugopalan P.L., De Ávila B.E.-F., Pal M., Ghosh A., Wang J. (2020). Fantastic Voyage of Nanomotors into the Cell. ACS Nano.

[6] Wang B., Kostarelos K., Nelson B.J., Zhang L. (2021). Trends in Micro-/Nanorobotics: Materials Development, Actuation, Localization, and System Integration for Biomedical Applications. Adv. Mater..

[7] Yasa I.C., Ceylan H., Bozuyuk U., Wild A.-M., Sitti M. (2020). Elucidating the interaction dynamics between microswimmer body and immune system for medical microrobots. Sci. Robot..

[8] Hortelão A., Simó C., Guix M., Guallar-Garrido S., Julián E., Vilela D., Rejc L., Ramos-Cabrer P., Cossío U., Gómez-Vallejo V. (2021). Swarming behavior and in vivo monitoring of enzymatic nanomotors within the bladder. Sci. Robot..

[9] Soto F., Karshalev E., Zhang F., Esteban Fernandez de Avila B., Nourhani A., Wang J. (2021). Smart Materials for Microrobots. Chem. Rev..

[10] Mujtaba J., Liu J., Dey K.K., Li T., Chakraborty R., Xu K., Makarov D., Barmin R.A., Gorin D.A., Tolstoy V.P. (2021). Micro-Bio-Chemo-Mechanical-Systems: Micromotors, Microfluidics, and Nanozymes for Biomedical Applications. Adv. Mater..

[11] Cui J., Huang T.-Y., Luo Z., Testa P., Gu H., Chen X.-Z., Nelson B.J., Heyderman L.J. (2019). Nanomagnetic encoding of shape-morphing micromachines. Nature.

[12] Wu Z., Chen Y., Mukasa D., Pak O.S., Gao W. (2020). Medical micro/nanorobots in complex media. Chem. Soc. Rev..

[13] Schmidt C.K., Medina-Sánchez M., Edmondson R.J., Schmidt O.G. (2020). Engineering microrobots for targeted cancer therapies from a medical perspective. Nat. Commun..

[14] Rao K.J., Li F., Meng L., Zheng H., Cai F., Wang W. (2015). A Force to Be Reckoned with: A Review of Synthetic Microswimmers Powered by Ultrasound. Small.

[15] Xu L., Mou F., Gong H., Luo M., Guan J. (2017). Light-driven micro/nanomotors: From fundamentals to applications. Chem. Soc. Rev..

[16] Chen X.-Z., Hoop M., Mushtaq F., Siringil E., Hu C., Nelson B.J., Pané S. (2017). Recent developments in magnetically driven micro- and nanorobots. Appl. Mater. Today.

[17] Jurado-Sánchez B., Wang J. (2018). Micromotors for environmental applications: A review. Environ. Sci. Nano.

[18] Šípová-Jungová H., Andrén D., Jones S., Käll M. (2020). Nanoscale Inorganic Motors Driven by Light: Principles, Realizations, and Opportunities. Chem. Rev..

[19] Sánchez S., Soler L., Katuri J. (2015). Chemically Powered Micro- and Nanomotors. Angew. Chem. Int. Ed..

[20] Anderson J.L. (1989). Colloid Transport by Interfacial Forces. Annu. Rev. Fluid Mech..

[21] Paxton W.F., Kistler K.C., Olmeda C.C., Sen A., Angelo S.K.S., Cao Y., Mallouk T.E., Lammert P.E., Crespi V.H. (2004). Catalytic Nanomotors:  Autonomous Movement of Striped Nanorods. J. Am. Chem. Soc..

[22] Yamamoto D., Takada T., Tachibana M., Iijima Y., Shioi A., Yoshikawa K. (2015). Micromotors working in water through artificial aerobic metabolism. Nanoscale.

[23] Gao W., D’Agostino M., Garcia-Gradilla V., Orozco J., Wang J. (2013). Multi-Fuel Driven Janus Micromotors. Small.

[24] Agrawal A., Dey K.K., Paul A., Basu S., Chattopadhyay A. (2008). Chemical Locomotives Based on Polymer Supported Catalytic Nanoparticles. J. Phys. Chem. C.

[25] Pinchasik B.-E., Möhwald H., Skirtach A.G. (2014). Mimicking Bubble Use in Nature: Propulsion of Janus Particles due to Hydrophobic-Hydrophilic Interactions. Small.

[26] Gao W., Pei A., Dong R., Wang J. (2014). Catalytic Iridium-Based Janus Micromotors Powered by Ultralow Levels of Chemical Fuels. J. Am. Chem. Soc..

[27] Gao W., Uygun A., Wang J. (2012). Hydrogen-Bubble-Propelled Zinc-Based Microrockets in Strongly Acidic Media. J. Am. Chem. Soc..

[28] Gao W., Pei A., Wang J. (2012). Water-Driven Micromotors. ACS Nano.

[29] Gao W., Feng X., Pei A., Gu Y., Li J., Wang J. (2013). Seawater-driven magnesium based Janus micromotors for environmental remediation. Nanoscale.

[30] Liu R., Sen A. (2011). Autonomous Nanomotor Based on Copper–Platinum Segmented Nanobattery. J. Am. Chem. Soc..

[31] Guix M., Meyer A.K., Koch B., Schmidt O.G. (2016). Carbonate-based Janus micromotors moving in ultra-light acidic environment generated by HeLa cells in situ. Sci. Rep..

[32] Moran J.L., Posner J.D. (2017). Phoretic Self-Propulsion. Annu. Rev. Fluid Mech..

[33] Cordova-Figueroa U.M., Brady J.F. (2008). Osmotic propulsion: The osmotic motor. Phys. Rev. Lett..

[34] Prieve D., Anderson J., Ebel J., Lowell M. (1984). Motion of a particle generated by chemical gradients. Part 2. Electrolytes. J. Fluid Mech..

[35] Sharifi-Mood N., Koplik J., Maldarelli C. (2013). Diffusiophoretic self-propulsion of colloids driven by a surface reaction: The sub-micron particle regime for exponential and van der Waals interactions. Phys. Fluids.

[36] Ebbens S., Gregory D.A., Dunderdale G., Howse J.R., Ibrahim Y., Liverpool T.B., Golestanian R. (2014). Electrokinetic effects in catalytic platinum-insulator Janus swimmers. EPL.

[37] Brown A., Poon W. (2014). Ionic effects in self-propelled Pt-coated Janus swimmers. Soft Matter.

[38] Howse J.R., Jones R.A.L., Ryan A.J., Gough T., Vafabakhsh R., Golestanian R. (2007). Self-Motile Colloidal Particles: From Directed Propulsion to Random Walk. Phys. Rev. Lett..

[39] Ke H., Ye S., Carroll R.L., Showalter K. (2010). Motion Analysis of Self-Propelled Pt−Silica Particles in Hydrogen Peroxide Solutions. J. Phys. Chem. A.

[40] Lee T.-C., Alarcón-Correa M., Miksch C., Hahn K., Gibbs J.G., Fischer P. (2014). Self-Propelling Nanomotors in the Presence of Strong Brownian Forces. Nano Lett..

[41] Ebbens S., Tu M.-H., Howse J.R., Golestanian R. (2012). Size dependence of the propulsion velocity for catalytic Janus-sphere swimmers. Phys. Rev. E.

[42] Ebbens S.J., Howse J.R. (2011). Direct Observation of the Direction of Motion for Spherical Catalytic Swimmers. Langmuir.

[43] Dong R., Zhang Q., Gao W., Pei A., Ren B. (2016). Highly Efficient Light-Driven TiO2–Au Janus Micromotors. ACS Nano.

[44] Zhou C., Chen X., Han Z., Wang W. (2019). Photochemically Excited, Pulsating Janus Colloidal Motors of Tunable Dynamics. ACS Nano.

[45] Wang X., Baraban L., Nguyen A., Ge J., Misko V.R., Tempere J., Nori F., Formanek P., Huang T., Cuniberti G. (2018). High-Motility Visible Light-Driven Ag/AgCl Janus Micromotors. Small.

[46] Chen C., Tang S., Teymourian H., Karshalev E., Zhang F., Li J., Mou F., Liang Y., Guan J., Wang J. (2018). Chemical/Light-Powered Hybrid Micromotors with “On-the-Fly” Optical Brakes. Angew. Chem. Int. Ed..

[47] Pavlick R.A., Sengupta S., McFadden T., Zhang H., Sen A. (2011). A Polymerization-Powered Motor. Angew. Chem. Int. Ed..

[48] Moran J.L., Wheat P.M., Posner J.D. (2010). Locomotion of electrocatalytic nanomotors due to reaction induced charge autoelectrophoresis. Phys. Rev. E.

[49] Nourhani A., Crespi V.H., Lammert P.E., Borhan A. (2015). Self-electrophoresis of spheroidal electrocatalytic swimmers. Phys. Fluids.

[50] Sabass B., Seifert U. (2012). Nonlinear, electrocatalytic swimming in the presence of salt. J. Chem. Phys..

[51] Gibbs J.G., Zhao Y.-P. (2009). Autonomously motile catalytic nanomotors by bubble propulsion. Appl. Phys. Lett..

[52] Li J., Huang G., Ye M., Li M., Liu R., Mei Y. (2011). Dynamics of catalytic tubular microjet engines: Dependence on geometry and chemical environment. Nanoscale.

[53] Gallino G., Gallaire F., Lauga E., Michelin S. (2018). Physics of Bubble-Propelled Microrockets. Adv. Funct. Mater..

[54] Mou F., Kong L., Chen C., Chen Z., Xu L., Guan J. (2016). Light-controlled propulsion, aggregation and separation of water-fuelled TiO2/Pt Janus submicromotors and their “on-the-fly” photocatalytic activities. Nanoscale.

[55] Zhang Q., Dong R., Wu Y., Gao W., He Z., Ren B. (2017). Light-Driven Au-WO3@C Janus Micromotors for Rapid Photodegradation of Dye Pollutants. ACS Appl. Mater. Interfaces.

[56] Pourrahimi A.M., Villa K., Manzanares Palenzuela C.L., Ying Y., Sofer Z., Pumera M. (2019). Catalytic and Light-Driven ZnO/Pt Janus Nano/Micromotors: Switching of Motion Mechanism via Interface Roughness and Defect Tailoring at the Nanoscale. Adv. Funct. Mater..

[57] Dong R., Hu Y., Wu Y., Gao W., Ren B., Wang Q., Cai Y. (2017). Visible-Light-Driven BiOI-Based Janus Micromotor in Pure Water. J. Am. Chem. Soc..

[58] Duhr S., Braun D. (2006). Thermophoretic Depletion Follows Boltzmann Distribution. Phys. Rev. Lett..

[59] Jiang H.-R., Yoshinaga N., Sano M. (2010). Active Motion of a Janus Particle by Self-Thermophoresis in a Defocused Laser Beam. Phys. Rev. Lett..

[60] Maggi C., Saglimbeni F., Dipalo M., De Angelis F., Di Leonardo R. (2015). Micromotors with asymmetric shape that efficiently convert light into work by thermocapillary effects. Nat. Commun..

[61] Rikken R.S.M., Nolte R.J.M., Maan J.C., van Hest J.C.M., Wilson D.A., Christianen P.C.M. (2014). Manipulation of micro-and nanostructure motion with magnetic fields. Soft Matter.

[62] Liu M., Wu F., Piao H., Huang X., Cong J., Luo Z., Pan L., Liu Y. (2017). Rod-shaped nanomotor powered by magnetic field gradients and its application to surface-enhanced Raman-scattering-based detection. Appl. Phys. Express.

[63] Baraban L., Makarov D., Streubel R., Mönch I., Grimm D., Sanchez S., Schmidt O.G. (2012). Catalytic Janus Motors on Microfluidic Chip: Deterministic Motion for Targeted Cargo Delivery. ACS Nano.

[64] Kline T.R., Paxton W.F., Mallouk T.E., Sen A. (2005). Catalytic Nanomotors: Remote-Controlled Autonomous Movement of Striped Metallic Nanorods. Angew. Chem. Int. Ed..

[65] Ahmed S., Wang W., Mair L.O., Fraleigh R.D., Li S., Castro L.A., Hoyos M., Huang T.J., Mallouk T.E. (2013). Steering Acoustically Propelled Nanowire Motors toward Cells in a Biologically Compatible Environment Using Magnetic Fields. Langmuir.

[66] Zhang L., Abbott J.J., Dong L., Kratochvil B.E., Bell D., Nelson B.J. (2009). Artificial bacterial flagella: Fabrication and magnetic control. Appl. Phys. Lett..

[67] Pal M., Somalwar N., Singh A., Bhat R., Eswarappa S.M., Saini D.K., Ghosh A. (2018). Maneuverability of Magnetic Nanomotors Inside Living Cells. Adv. Mater..

[68] Gao W., Sattayasamitsathit S., Manesh K.M., Weihs D., Wang J. (2010). Magnetically Powered Flexible Metal Nanowire Motors. J. Am. Chem. Soc..

[69] Cheang U.K., Roy D., Lee J.H., Kim M.J. (2010). Fabrication and magnetic control of bacteria-inspired robotic microswimmers. Appl. Phys. Lett..

[70] Purcell E.M. (1977). Life at low Reynolds number. Am. J. Phys..

[71] Jang B., Gutman E., Stucki N., Seitz B.F., Wendel-García P.D., Newton T., Pokki J., Ergeneman O., Pané S., Or Y. (2015). Undulatory Locomotion of Magnetic Multilink Nanoswimmers. Nano Lett..

[72] Wang W., Castro L.A., Hoyos M., Mallouk T.E. (2012). Autonomous Motion of Metallic Microrods Propelled by Ultrasound. ACS Nano.

[73] Soto F., Wagner G.L., Garcia-Gradilla V., Gillespie K.T., Lakshmipathy D.R., Karshalev E., Angell C., Chen Y., Wang J. (2016). Acoustically propelled nanoshells. Nanoscale.

[74] Bruus H. (2012). Acoustofluidics 7: The acoustic radiation force on small particles. Lab Chip.

[75] Ahmed D., Baasch T., Jang B., Pane S., Dual J., Nelson B.J. (2016). Artificial Swimmers Propelled by Acoustically Activated Flagella. Nano Lett..

[76] Xuan M., Shao J., Lin X., Dai L., He Q. (2014). Self-Propelled Janus Mesoporous Silica Nanomotors with Sub-100 nm Diameters for Drug Encapsulation and Delivery. ChemPhysChem.

[77] Orozco J., Mercante L.A., Pol R., Merkoçi A. (2016). Graphene-based Janus micromotors for the dynamic removal of pollutants. J. Mater. Chem. A.

[78] Choi H., Cho S.H., Hahn S.K. (2020). Urease-Powered Polydopamine Nanomotors for Intravesical Therapy of Bladder Diseases. ACS Nano.

[79] Wan M., Wang Q., Wang R., Wu R., Li T., Fang D., Huang Y., Yu Y., Fang L., Wang X. (2020). Platelet-derived porous nanomotor for thrombus therapy. Sci. Adv..

[80] Wang S., Wu N. (2014). Selecting the Swimming Mechanisms of Colloidal Particles: Bubble Propulsion versus Self-Diffusiophoresis. Langmuir.

[81] Choudhury U., Soler L., Gibbs J.G., Sanchez S., Fischer P. (2015). Surface roughness-induced speed increase for active Janus micromotors. Chem. Commun..

[82] Wu Y., Wu Z., Lin X., He Q., Li J. (2012). Autonomous Movement of Controllable Assembled Janus Capsule Motors. ACS Nano.

[83] Longbottom B.W., Bon S.A.F. (2018). Improving the engine power of a catalytic Janus-sphere micromotor by roughening its surface. Sci. Rep..

[84] Li Y., Mou F., Chen C., You M., Yin Y., Xu L., Guan J. (2016). Light-controlled bubble propulsion of amorphous TiO_2_/Au Janus micromotors. RSC Adv..

[85] Ma X., Hortelão A.C., Patiño T., Sánchez S. (2016). Enzyme Catalysis to Power Micro/Nanomachines. ACS Nano.

[86] Joseph A., Contini C., Cecchin D., Nyberg S., Ruiz-Perez L., Gaitzsch J., Fullstone G., Tian X., Azizi J., Preston J. (2017). Chemotactic synthetic vesicles: Design and applications in blood-brain barrier crossing. Sci. Adv..

[87] Uygun D.A., Jurado-Sánchez B., Uygun M., Wang J. (2016). Self-propelled chelation platforms for efficient removal of toxic metals. Environ. Sci. Nano.

[88] Vilela D., Stanton M.M., Parmar J., Sánchez S. (2017). Microbots Decorated with Silver Nanoparticles Kill Bacteria in Aqueous Media. Acs Appl. Mater. Interfaces.

[89] Mou F., Chen C., Ma H., Yin Y., Wu Q., Guan J. (2013). Self-Propelled Micromotors Driven by the Magnesium–Water Reaction and Their Hemolytic Properties. Angew. Chem. Int. Ed..

[90] Chen C., Karshalev E., Li J., Soto F., Castillo R., Campos I., Mou F., Guan J., Wang J. (2016). Transient Micromotors That Disappear When No Longer Needed. ACS Nano.

[91] Saad S., Kaur H., Natale G. (2020). Scalable Chemical Synthesis Route to Manufacture pH-Responsive Janus CaCO3 Micromotors. Langmuir.

[92] Zhang H., Duan W., Liu L., Sen A. (2013). Depolymerization-Powered Autonomous Motors Using Biocompatible Fuel. J. Am. Chem. Soc..

[93] Xuan M., Shao J., Gao C., Wang W., Dai L., He Q. (2018). Self-Propelled Nanomotors for Thermomechanically Percolating Cell Membranes. Angew. Chem. Int. Ed..

[94] Xuan M., Wu Z., Shao J., Dai L., Si T., He Q. (2016). Near Infrared Light-Powered Janus Mesoporous Silica Nanoparticle Motors. J. Am. Chem. Soc..

[95] Qian B., Montiel D., Bregulla A., Cichos F., Yang H. (2013). Harnessing thermal fluctuations for purposeful activities: The manipulation of single micro-swimmers by adaptive photon nudging. Chem. Sci..

[96] Wang Y., Hernandez R.M., Bartlett D.J., Bingham J.M., Kline T.R., Sen A., Mallouk T.E. (2006). Bipolar Electrochemical Mechanism for the Propulsion of Catalytic Nanomotors in Hydrogen Peroxide Solutions. Langmuir.

[97] Sundararajan S., Sengupta S., Ibele M.E., Sen A. (2010). Drop-Off of Colloidal Cargo Transported by Catalytic Pt–Au Nanomotors via Photochemical Stimuli. Small.

[98] Sundararajan S., Lammert P.E., Zudans A.W., Crespi V.H., Sen A. (2008). Catalytic Motors for Transport of Colloidal Cargo. Nano Lett..

[99] Laocharoensuk R., Burdick J., Wang J. (2008). Carbon-Nanotube-Induced Acceleration of Catalytic Nanomotors. ACS Nano.

[100] Chen B., Liu L., Liu K., Tong F., Wang S., Fu D., Gao J., Jiang J., Ou J., Ye Y. (2020). Photoelectrochemical TiO_2_-Au-Nanowire-Based Motor for Precise Modulation of Single-Neuron Activities. Adv. Funct. Mater..

[101] Wang J., Xiong Z., Zhan X., Dai B., Zheng J., Liu J., Tang J. (2017). A Silicon Nanowire as a Spectrally Tunable Light-Driven Nanomotor. Adv. Mater..

[102] Mirkovic T., Foo M.L., Arsenault A.C., Fournier-Bidoz S., Zacharia N.S., Ozin G.A. (2007). Hinged nanorods made using a chemical approach to flexible nanostructures. Nat. Nanotechnol..

[103] Li T., Li J., Zhang H., Chang X., Song W., Hu Y., Shao G., Sandraz E., Zhang G., Li L. (2016). Magnetically Propelled Fish-Like Nanoswimmers. Small.

[104] Liu Y., Ge D., Cong J., Piao H.-G., Huang X., Xu Y., Lu G., Pan L., Liu M. (2018). Magnetically Powered Annelid-Worm-Like Microswimmers. Small.

[105] Dreyfus R., Baudry J., Roper M.L., Fermigier M., Stone H.A., Bibette J. (2005). Microscopic artificial swimmers. Nature.

[106] Gao W., Manesh K.M., Hua J., Sattayasamitsathit S., Wang J. (2011). Hybrid Nanomotor: A Catalytically/Magnetically Powered Adaptive Nanowire Swimmer. Small.

[107] Zhang L., Petit T., Peyer K., Nelson B.J. Nickel nanowire swimmers for colloidal cargo transport near a solid surface. Proceedings of the 11th IEEE International Conference on Nanotechnology.

[108] Garcia-Gradilla V., Orozco J., Sattayasamitsathit S., Soto F., Kuralay F., Pourazary A., Katzenberg A., Gao W., Shen Y., Wang J. (2013). Functionalized Ultrasound-Propelled Magnetically Guided Nanomotors: Toward Practical Biomedical Applications. ACS Nano.

[109] Nadal F., Lauga E. (2014). Asymmetric steady streaming as a mechanism for acoustic propulsion of rigid bodies. Phys. Fluids.

[110] Hansen-Bruhn M., de Ávila B.E.-F., Beltrán-Gastélum M., Zhao J., Ramírez-Herrera D.E., Angsantikul P., Vesterager Gothelf K., Zhang L., Wang J. (2018). Active Intracellular Delivery of a Cas9/sgRNA Complex Using Ultrasound-Propelled Nanomotors. Angew. Chem. Int. Ed..

[111] De Ávila B.E.-F., Martín A., Soto F., Lopez-Ramirez M.A., Campuzano S., Vásquez-Machado G.M., Gao W., Zhang L., Wang J. (2015). Single Cell Real-Time miRNAs Sensing Based on Nanomotors. ACS Nano.

[112] Beltrán-Gastélum M., De Ávila B.E.-F., Gong H., Venugopalan P.L., Hianik T., Wang J., Subjakova V. (2019). Rapid Detection of AIB1 in Breast Cancer Cells Based on Aptamer-Functionalized Nanomotors. ChemPhysChem.

[113] Solovev A.A., Mei Y., Bermúdez Ureña E., Huang G., Schmidt O.G. (2009). Catalytic Microtubular Jet Engines Self-Propelled by Accumulated Gas Bubbles. Small.

[114] Manjare M., Yang B., Zhao Y.P. (2013). Bubble-Propelled Microjets: Model and Experiment. J. Phys. Chem. C.

[115] Li L., Wang J., Li T., Song W., Zhang G. (2014). Hydrodynamics and propulsion mechanism of self-propelled catalytic micromotors: Model and experiment. Soft Matter.

[116] Fomin V.M., Hippler M., Magdanz V., Soler L., Sanchez S., Schmidt O.G. (2014). Propulsion Mechanism of Catalytic Microjet Engines. IEEE Trans. Robot. A Publ. IEEE Robot. Autom. Soc..

[117] Enachi M., Guix M., Postolache V., Ciobanu V., Fomin V.M., Schmidt O.G., Tiginyanu I. (2016). Light-Induced Motion of Microengines Based on Microarrays of TiO_2_ Nanotubes. Small.

[118] Kagan D., Benchimol M.J., Claussen J.C., Chuluun-Erdene E., Esener S., Wang J. (2012). Acoustic Droplet Vaporization and Propulsion of Perfluorocarbon-Loaded Microbullets for Targeted Tissue Penetration and Deformation. Angew. Chem. Int. Ed..

[119] Wilson D.A., Nolte R.J.M., van Hest J.C.M. (2012). Autonomous movement of platinum-loaded stomatocytes. Nat. Chem..

[120] Abdelmohsen L.K.E.A., Nijemeisland M., Pawar G.M., Janssen G.-J.A., Nolte R.J.M., van Hest J.C.M., Wilson D.A. (2016). Dynamic Loading and Unloading of Proteins in Polymeric Stomatocytes: Formation of an Enzyme-Loaded Supramolecular Nanomotor. ACS Nano.

[121] Tu Y., Peng F., White P.B., Wilson D.A. (2017). Redox-Sensitive Stomatocyte Nanomotors: Destruction and Drug Release in the Presence of Glutathione. Angew. Chem. Int. Ed..

[122] Tu Y., Peng F., Sui X., Men Y., White P.B., van Hest J.C.M., Wilson D.A. (2017). Self-propelled supramolecular nanomotors with temperature-responsive speed regulation. Nat. Chem..

[123] Xuan M., Mestre R., Gao C., Zhou C., He Q., Sánchez S. (2018). Noncontinuous Super-Diffusive Dynamics of a Light-Activated Nanobottle Motor. Angew. Chem. Int. Ed..

[124] Zhou C., Gao C., Lin Z., Wang D., Li Y., Yuan Y., Zhu B., He Q. (2020). Autonomous Motion of Bubble-Powered Carbonaceous Nanoflask Motors. Langmuir.

[125] Yi D., Zhang Q., Liu Y., Song J., Tang Y., Caruso F., Wang Y. (2016). Synthesis of Chemically Asymmetric Silica Nanobottles and Their Application for Cargo Loading and as Nanoreactors and Nanomotors. Angew. Chem. Int. Ed..

[126] Jiang S., Kaltbeitzel A., Hu M., Suraeva O., Crespy D., Landfester K. (2020). One-Step Preparation of Fuel-Containing Anisotropic Nanocapsules with Stimuli-Regulated Propulsion. ACS Nano.

[127] Lauga E., Powers T.R. (2009). The hydrodynamics of swimming microorganisms. Rep. Prog. Phys..

[128] Walker D., Käsdorf B.T., Jeong H.-H., Lieleg O., Fischer P. (2015). Enzymatically active biomimetic micropropellers for the penetration of mucin gels. Sci. Adv..

[129] Yu Y., Shang L., Gao W., Zhao Z., Wang H., Zhao Y. (2017). Microfluidic Lithography of Bioinspired Helical Micromotors. Angew. Chem. Int. Ed..

[130] Di Leonardo R., Angelani L., Dell’Arciprete D., Ruocco G., Iebba V., Schippa S., Conte M.P., Mecarini F., De Angelis F., Di Fabrizio E. (2010). Bacterial ratchet motors. Proc. Natl. Acad. Sci. USA.

[131] Sokolov A., Apodaca M.M., Grzybowski B.A., Aranson I.S. (2010). Swimming bacteria power microscopic gears. Proc. Natl. Acad. Sci. USA.

[132] Brooks A.M., Tasinkevych M., Sabrina S., Velegol D., Sen A., Bishop K.J.M. (2019). Shape-directed rotation of homogeneous micromotors via catalytic self-electrophoresis. Nat. Commun..

[133] Carlotti M., Mattoli V. (2019). Functional Materials for Two-Photon Polymerization in Microfabrication. Small.

[134] Rus D., Tolley M.T. (2018). Design, fabrication and control of origami robots. Nat. Rev. Mater..

[135] Zeng H., Wasylczyk P., Parmeggiani C., Martella D., Burresi M., Wiersma D.S. (2015). Light-Fueled Microscopic Walkers. Adv. Mater..

[136] McNeill J.M., Nama N., Braxton J.M., Mallouk T.E. (2020). Wafer-Scale Fabrication of Micro- to Nanoscale Bubble Swimmers and Their Fast Autonomous Propulsion by Ultrasound. ACS Nano.

[137] Ren L.Q., Nama N., McNeill J.M., Soto F., Yan Z.F., Liu W., Wang W., Wang J., Mallouk T.E. (2019). 3D steerable, acoustically powered microswimmers for single-particle manipulation. Sci. Adv..

[138] Aghakhani A., Yasa O., Wrede P., Sitti M. (2020). Acoustically powered surface-slipping mobile microrobots. Proc. Natl. Acad. Sci. USA.

[139] Miskin M.Z., Cortese A.J., Dorsey K., Esposito E.P., Reynolds M.F., Liu Q., Cao M., Muller D.A., McEuen P.L., Cohen I. (2020). Electronically integrated, mass-manufactured, microscopic robots. Nature.

[140] Huang T.-Y., Sakar M.S., Mao A., Petruska A.J., Qiu F., Chen X.-B., Kennedy S., Mooney D., Nelson B.J. (2015). 3D Printed Microtransporters: Compound Micromachines for Spatiotemporally Controlled Delivery of Therapeutic Agents. Adv. Mater..

[141] Koepele C.A., Guix M., Bi C., Adam G., Cappelleri D.J. (2020). 3D-Printed Microrobots with Integrated Structural Color for Identification and Tracking. Adv. Intell. Syst..

[142] Vilela D., Parmar J., Zeng Y., Zhao Y., Sánchez S. (2016). Graphene-Based Microbots for Toxic Heavy Metal Removal and Recovery from Water. Nano Lett..

[143] Mushtaq F., Asani A., Hoop M., Chen X.-Z., Ahmed D., Nelson B.J., Pané S. (2016). Highly Efficient Coaxial TiO_2_-PtPd Tubular Nanomachines for Photocatalytic Water Purification with Multiple Locomotion Strategies. Adv. Funct. Mater..

[144] Kochergin Y.S., Villa K., Novotný F., Plutnar J., Bojdys M.J., Pumera M. (2020). Multifunctional Visible-Light Powered Micromotors Based on Semiconducting Sulfur-and Nitrogen-Containing Donor–Acceptor Polymer. Adv. Funct. Mater..

[145] Mou F., Pan D., Chen C., Gao Y., Xu L., Guan J. (2015). Magnetically Modulated Pot-Like MnFe_2_O_4_ Micromotors: Nanoparticle Assembly Fabrication and their Capability for Direct Oil Removal. Adv. Funct. Mater..

[146] Delezuk J.A.M., Ramírez-Herrera D.E., De Ávila B.E.-F., Wang J. (2017). Chitosan-based water-propelled micromotors with strong antibacterial activity. Nanoscale.

[147] Singh V.V., Jurado-Sánchez B., Sattayasamitsathit S., Orozco J., Li J., Galarnyk M., Fedorak Y., Wang J. (2015). Multifunctional Silver-Exchanged Zeolite Micromotors for Catalytic Detoxification of Chemical and Biological Threats. Adv. Funct. Mater..

[148] Campuzano S., Orozco J., Kagan D., Guix M., Gao W., Sattayasamitsathit S., Claussen J.C., Merkoçi A., Wang J. (2012). Bacterial Isolation by Lectin-Modified Microengines. Nano Lett..

[149] Kiristi M., Singh V.V., De Ávila B.E.-F., Uygun M., Soto F., Aktaş Uygun D., Wang J. (2015). Lysozyme-Based Antibacterial Nanomotors. ACS Nano.

[150] De Ávila B.E.-F., Angsantikul P., Li J., Angel Lopez-Ramirez M., Ramírez-Herrera D.E., Thamphiwatana S., Chen C., Delezuk J., Samakapiruk R., Ramez V. (2017). Micromotor-enabled active drug delivery for in vivo treatment of stomach infection. Nat. Commun..

[151] Hortelão A.C., Patiño T., Perez-Jiménez A., Blanco À., Sánchez S. (2018). Enzyme-Powered Nanobots Enhance Anticancer Drug Delivery. Adv. Funct. Mater..

[152] Wang D., Gao C., Wang W., Sun M., Guo B., Xie H., He Q. (2018). Shape-Transformable, Fusible Rodlike Swimming Liquid Metal Nanomachine. ACS Nano.

[153] Chen X.-Z., Hoop M., Shamsudhin N., Huang T., Özkale B., Li Q., Siringil E., Mushtaq F., Di Tizio L., Nelson B.J. (2017). Hybrid Magnetoelectric Nanowires for Nanorobotic Applications: Fabrication, Magnetoelectric Coupling, and Magnetically Assisted In Vitro Targeted Drug Delivery. Adv. Mater..

[154] Hortelão A.C., Carrascosa R., Murillo-Cremaes N., Patiño T., Sánchez S. (2019). Targeting 3D Bladder Cancer Spheroids with Urease-Powered Nanomotors. ACS Nano.

[155] Sun L., Yu Y., Chen Z., Bian F., Ye F., Sun L., Zhao Y. (2020). Biohybrid robotics with living cell actuation. Chem. Soc. Rev..

[156] Singh A.V., Ansari M.H.D., Mahajan M., Srivastava S., Kashyap S., Dwivedi P., Pandit V., Katha U. (2020). Sperm Cell Driven Microrobots-Emerging Opportunities and Challenges for Biologically Inspired Robotic Design. Micromachines.

[157] Felfoul O., Mohammadi M., Taherkhani S., de Lanauze D., Zhong Xu Y., Loghin D., Essa S., Jancik S., Houle D., Lafleur M. (2016). Magneto-aerotactic bacteria deliver drug-containing nanoliposomes to tumour hypoxic regions. Nat. Nanotechnol..

[158] Mestre R., Patiño T., Sánchez S. (2021). Biohybrid robotics: From the nanoscale to the macroscale. WIREs Nanomed. Nanobiotechnol..

